# The Latest Data Specifically Focused on Long-Term Oncologic Prognostication for Very Old Adults with Acute Vulnerable Localized Prostate Cancer: A Nationwide Cohort Study

**DOI:** 10.3390/jcm11123451

**Published:** 2022-06-15

**Authors:** Szu-Yuan Wu, Fransisca Fortunata Effendi, Ricardo E. Canales, Chung-Chien Huang

**Affiliations:** 1 Department of Food Nutrition and Health Biotechnology, College of Medical and Health Science, Asia University, Taichung 413, Taiwan; szuyuanwu5399@gmail.com; 2 Big Data Center, Lo-Hsu Medical Foundation, Lotung Poh-Ai Hospital, Yilan 265, Taiwan; 3 Division of Radiation Oncology, Lo-Hsu Medical Foundation, Lotung Poh-Ai Hospital, Yilan 265, Taiwan; 4 Department of Healthcare Administration, College of Medical and Health Science, Asia University, Taichung 413, Taiwan; 5 Cancer Center, Lo-Hsu Medical Foundation, Lotung Poh-Ai Hospital, Yilan 265, Taiwan; 6 Graduate Institute of Business Administration, Fu Jen Catholic University, Taipei 242, Taiwan; 7 Centers for Regional Anesthesia and Pain Medicine, Taipei Municipal Wan Fang Hospital, Taipei Medical University, Taipei 110, Taiwan; 8 School of Health Care Administration, Department of Health Care Administration, College of Management, Taipei Medical University, Taipei 110, Taiwan; m911109016@tmu.edu.tw (F.F.E.); recanalesmd@gmail.com (R.E.C.); 9 PT Inertia Utama, Dexa Group, Jl. Boulevard Bintaro Jaya, Pondok Jaya, Pondok Aren, South Tangerang 15117, Indonesia; 10 Collegiate of Doctors in Honduras, Colegio Médico de Honduras (CMH), CA 6 Boulevard Fuerzas Armadas, Tegucigalpa 11101, Honduras; 11 Biotech and Healthcare Management, School of Health Care Administration, College of Management, Taipei Medical University, Taipei 110, Taiwan; 12 Department of Medical Quality, Taipei Municipal Wan Fang Hospital, Taipei Medical University, Taipei 110, Taiwan; 13 Department of Long-Term Care & School of Gerontology Health Management, College of Nursing, Taipei Medical University, Taipei 110, Taiwan; 14 Department & School of Pharmacy, College of Pharmacy, Taipei Medical University, Taipei 110, Taiwan

**Keywords:** prostate cancer, acute vulnerable, radical prostatectomy, intensity-modulated radiotherapy, oncologic prognostication

## Abstract

Purpose: Few studies have evaluated the prime treatment choice for men older than 80 years with acute vulnerable localized prostate cancer (AV-LPC). Clinicians have been deeply troubled by this near end-of-life medical choice for a very specific group of patients. We compared the oncological prognostication of very old patients with AV-LPC after they underwent either radical prostatectomy (RP) or massive-dose intensity-modulated radiotherapy (IMRT) coupled with long-term androgen deprivation therapy (ADT) over a long-term investigation. Methods: In this nationwide cohort study, we used the Taiwan Cancer Registry Database and retrieved information related to patients (aged ≥ 80 years) with AV-LPC who underwent standard RP (the RP group) or massive-dose IMRT + long-term ADT (at least 72 Gy and ADT use ≥18 months; the IMRT + ADT group). After potential confounders were controlled for using propensity score matching (PSM), we utilized the Cox proportional hazards regression to evaluate the oncologic prognostication. Results: The IMRT + ADT group had a significantly higher adjusted hazard ratio (aHR) for all-cause mortality (aHR, 2.00; 95% confidence interval [CI], 1.41–2.87) than the RP group. Analysis of the secondary outcomes revealed that compared with the RP group, the aHRs of biochemical failure, locoregional recurrence, and distant metastasis in the IMRT + ADT group were 1.77 (95% CI: 1.36–2.11, *p* < 0.0001), 1.12 (95% CI: 1.04–1.33, *p* < 0.0001), and 1.15 (95% CI: 1.06–1.71, *p* = 0.0311), respectively. Conclusion: RP provides more favorable oncological prognostication than IMRT in very old adults with AV-LPC.

## 1. Introduction

Prostate cancer (PC) is the second leading cause of cancer in men, with an incidence of nearly 1.3 million in 2018 [1,2], and the fifth most prevalent cancer worldwide [3]. Its incidence has been increasing in many developed countries, including those in Europe and the Americas, as well as in Australia, New Zealand, and Taiwan [4,5,6]. PC is commonly diagnosed in older men (i.e., ≥70 years old). As the population continues to age and life expectancy continues to increase, particularly in developed countries, PC incidence is predicted to rise to 2.3 million cases in 2040 [1,7]. This indicates that clinicians will encounter an increasing number of older adults with PC [8,9,10]. Therefore, choosing the prime curative-intent treatments in older men with PC has become increasingly crucial in geriatric and oncological medicine.

Many risk classifications exist for PC, such as those proposed by EAU et al., the American Urological Association (AUA), the European Association of Urology (EAU), and the National Comprehensive Cancer Network (NCCN) [11]. Factors considered for such classification include prostate-specific antigen (PSA), biopsy Gleason score (GS), and clinical T stage. Those factors are applied to categorize patients and guide treatment modality decision making [12,13,14]. Therefore, many studies have evaluated the risk classifications of PC in terms of management [12,13,14]. Treatments for PC with inconsistent risk classifications in previous studies might be not reasonably feasible and unsuitably extrapolated to other treatments with different risk classifications [12,13,14]. Men who have acute vulnerable localized PC (AV-LPC), as diagnosed using the NCCN risk classification, exhibit favorable response to localized curative therapy [15]. AV-LPC is defined as the presence of clinical T stage ≥ T3a, GS ≥ 8, or PSA > 20 mg/mL [15,16] and can lead to death if not suitably treated [17]. Patients with AV-LPC are commonly treated with radical prostatectomy (RP) or radiotherapy (RT) plus long-term androgen deprivation therapy (ADT) [11,18]. Studies comparing RP and RT in patients with LPC have yielded inconsistent results because of inconsistencies in patient ages and the use of different risk classifications, RT techniques, and ADT durations [19,20,21,22,23,24,25]. In Taiwan, LPC is treated only per NCCN guidelines and risk classification [4,5,6,26,27]. Previous studies have not been conducted to predict the oncologic prognostication of RP or RT for very old (≥80 years old) men with AV-LPC using NCCN risk groups. In this study, we evaluated RP or RT as the prime care treatment for older men with AV-LPC, as determined using NCCN criteria.

Due to the inconsistent results in previous studies [19,20,21,22,23,24,25], we used Taiwan’s National Health Insurance Research Database (NHIRD) to compare the long-term oncologic prognostication of two curative-intent treatments—RP (the RP group) and massive-dose IMRT + long-term ADT (the IMRT + ADT group)—based on all-cause mortality, biochemical failure (BF), locoregional recurrence, and distant metastasis (DM), by applying head-to-head propensity score matching (PSM). Our findings can serve as reference to clinicians when determining the prime treatment for very old patients with AV-LPC.

## 2. Methods

### 2.1. Database

For this retrospective study, data were obtained from the NHIRD, which is integrated with the Taiwan Cancer Registry Database (TCRD). Thanks to its integration with linking possibilities to other databases, the data associated with the treatment schemes and health care usage could be garnered together [6,26,28,29,30,31]. Thus, the database contained the necessary information, including comorbidities, treatments, medications, and Charlson comorbidity index (CCI) [6,26,28,29,30,31].

### 2.2. Study Cohort

From the TCRD, we retrieved the data of men aged ≥ 80 years with PC who had undergone RP or massive-dose IMRT + long-term ADT from 1 January 2008 to 31 December 2018. We specified the index date as the date PC diagnosis was performed. The diagnosis was assured through further assessment of pathological data and magnetic resonance images of PC clinical stages. The study protocols were probed and received approval from the Institutional Review Board of Tzu-Chi Medical Foundation (IRB109-015-B). The exclusion criteria included patients with different types of cancer, clinical lymph node metastasis, or any malignant neoplasms which have been classified based on the American Joint Committee on Cancer (AJCC), 7th edition, staging system. RP was defined as resection of the whole prostate gland and its peripheral lymph nodes [27]. Moreover, the standard IMRT protocol used in our study was as follows: prophylactic doses of 1.8–45 Gy per fraction to the pelvic lymph nodes and 54 Gy to the seminal vesicles and cone-down boosts of 72–81 Gy to cover the prostate (median dose: 75.6 Gy, median follow-up: 72.2 months). We excluded patients who received IMRT with scant doses (<72 Gy) or who had undergone <18 months of androgen deprivation therapy (ADT). Pathological confirmation of PC diagnosis was obtained through biopsy, which was used to decide whether the patient should undergo RP, RT, or active surveillance, based on the NCCN risk criteria and estimated patient survival time. We also excluded men with a history of cancer before PC diagnosis, unidentified clinical or pathological stage, unidentified EAU risk classification, unidentified GS, unidentified postoperative Gleason grade group, missing data on the pretreatment PSA concentration, clinical node-positive PC, and no adenocarcinoma histology. The scheme diagram description of the study is attached as Supplementary Figure S1.

### 2.3. Study Covariates

The study covariates comprised all factors related to mortality: therapy categorization, age, diagnosis year, wealth, hospital region, hospital tier (academic or nonacademic), clinical T stage, grade group (maximal Gleason grade), pretreatment PSA (ng/mL), and EAU risk classification. The comorbidities score was predicted by applying the CCI, in which the prominent comorbidities correlated with all-cause mortality. However, we merely incorporated comorbidities within six months prior to the index date. A comorbidity was included only if its International Classification of Diseases, Ninth Revision, Clinical Modification (ICD-9-CM) diagnostic code served as the main diagnosis for more than two visits to the outpatient department or first admission.

### 2.4. Endpoints

The main objective was the risk of all-cause mortality, and the subsidiary objectives were risk of BF, LRR, and DM. All endpoints were compared between the RP and IMRT + ADT groups.

### 2.5. Propensity Score Matching

The greedy method was utilized to match the cohorts with a ratio of 1:2, with aforesaid covariates in Supplemental Table S1 thoroughly matched with a propensity score within a caliper of 0.2 [32]. Cox proportional hazard curves were used to estimate all-cause mortality in patients receiving either treatment. Covariates in the RP group were 1:2, or 1:1 matched to those in the IMRT + ADT group through PSM with replacement, and all matched covariates in the RP and IMRT + ADT groups were included in the Cox proportional hazards model.

### 2.6. Statistics

The study model defined the duration from the index date to all-cause mortality. A Cox proportional hazards model was utilized to control for confounders. To lessen the influence of potential confounders, head-to-head PSM was used for between-group comparisons. All covariates in the massive-dose IMRT + ADT group were matched at a 1:2 ratio with those in the RP group through PSM. A strong and robust predictor was applied to account for clustering within matched sets, and a Cox model was applied to regress endpoints on treatment status. Subsequently, the multivariable Cox regression analysis was conducted to calculate the hazard ratios (HRs) to specify whether the covariates were necessitated to be readjusted to alleviate any confounding effects if an unbalanced condition existed after PSM. The tight control of potential prognosis factor was also performed during the analysis, and the endpoint was entire factors related to mortality in the treatment group.

The risk of all-cause mortality was enumerated for older men with AV-LPC. The additional subsidiary objectives, such as BF, LRR, and DM, were examined and predicted by utilizing a proportional sub-distribution hazard regression model to manage the competing risk of death in the analysis of time-to-event data. SAS software for Windows version 9.4 (SAS Institute Inc., Cary, NC, USA) was utilized to perform all statistical analyses. A *p* value of <0.05 derived using a two-tailed Wald test was considered statistically significant. The Kaplan–Meir method was utilized to predict the risk of all-cause mortality. Distinctions between IMRT + ADT or RP were specified by applying the stratified log-rank test to compare survival curves (stratified on matched sets).

## 3. Results

Of the 659 older men with AV-LPC, 277 and 382 received RP and IMRT + ADT, respectively (Supplementary Table S1). The mean follow-up duration for the RP and IMRT + long-term ADT groups was 61.7 ± 18.4 months and 58.4 ± 18.9 months, respectively. After PSM, no significant between-group differences were observed (*p* > 0.05) in the presence of the following covariates: age, year of diagnosis, CCI scores, major underlying diseases (myocardial infarction condition, congestive heart failure ailment, peripheral vascular related disorders, cerebrovascular problems, chronic pulmonary illness, high blood sugar, and high blood pressure), wealth, hospital region, hospital tier, clinical T stage, GS, grade group, PSA, and EAU risk classification. Most *p* values were close to 1, stipulating comparable disposition of the corresponding variables.

The treatment modality and age were significant estimators of all-cause death following multivariate Cox regression analysis (Table 1). Multivariable Cox regression analysis revealed that RP was associated with increased OS compared with IMRT + ADT in older patients with HR-PC. Because of PSM, no significant differences were presented in the controlled variables (Table 1), other than age. Compared with the RP group, the adjusted HR (aHR) (95% confidence interval [CI]) of the IMRT + ADT groups for all-cause mortality was 2.00 (1.41–2.87, *p* < 0.0001); BF, 1.77 (1.36–2.11, *p* < 0.0001) (Table 2); LRR, 1.12 (1.04–1.33, *p* < 0.0001) (Table 3); and DM, 1.15 (1.06–1.71, *p* = 0.0311) (Table 4).

**Figure 1:**
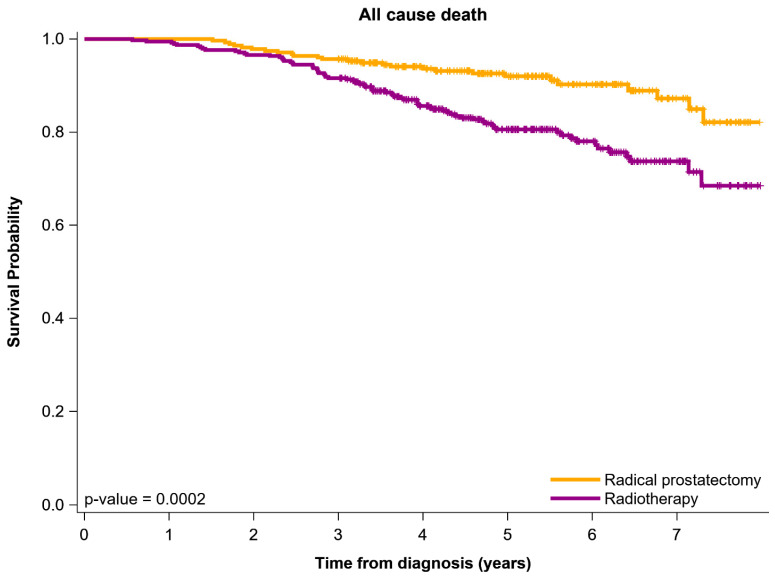
Plots of survival for outputs of patient with AV-LPC receiving different curative-intent treatments utilizing the Kaplan–Meier method: Propensity score-matching.

Kaplan–Meier OS curves of the two groups are displayed in Figure 1. The OS curve was more advanced for the RP group than for the IMRT + ADT group, with the 6-year OS rate being 92.1% and 79.8%, respectively.

## 4. Discussion

The prevalence of PC has increased to 75% in men aged > 70 years old [8]. Moreover, older patients have more aggressive PC in terms of higher risk of larger tumors, risk stratification, biochemical recurrence, distant metastasis, and disease-specific mortality [19,21]. Older age is a known risk factor that contributes to clinical decision making regarding treatment while considering life expectancy, comorbidity risk assessment, and postoperative complication risk. The final decision should consider several factors, including patient preference, treatment cost, quality of life, clinical staging, GS or grade group, pretreatment serum PSA level, and total of biopsy cores and cancer stage involvement [27,33,34]. The highest proportion of prostate cancer-specific mortality was found in men over 80 years old (19.59%) [35]. The watch-and-wait approach may thus be unsuitable for older men with AV-LPC. Accordingly, determination of the prime therapy (e.g., RP vs. IMRT + ADT) for these patients is crucial to impede disease progression [8,9].

Between RP and RT, RT is preferred for men older than 70 years [8,36,37], whereas conservative therapy such as watchful waiting or ADT (luteinizing hormone-releasing hormone analogs) is superior for men older than 80 years [8]. However, substantial measures should be taken for those patients as the most effective treatment due to poor clinical judgment to diagnose the interrelated issues in older patients with cancer [38]. RP and RT aim to minimize disease-specific mortality and its clinical progression, particularly compared with the active monitoring in men with AV-LPC, even in very old men. As suggested by the NCCN guidelines [11], RP may cause poorer outcomes in older patients because of weaker physical sustainability, more progressive disease, and increased pathological grades. Such recommendations have led to RT being preferred for men older than 75 years [19,20]. In our study, more older men with AV-LPC chose RT rather than RP (Supplementary Table S1), but no data exist to indicate that IMRT is superior to RP for older men with AV-LPC. We conducted the first research to address this issue.

Although older patients have a lower survival rate and higher complication rate than younger patients, age should not be the only criterion [39]. Notably, older men without any comorbidities have a lower risk of higher toxicity and thus higher tolerance to aggressive approaches [40]. In our study, approximately 70% of older men had CCI scores 0–1 (Supplemental Table S1), which means their life expectancy might be >5 years, worthy for more aggressive treatments, instead of active surveillance. However, prime therapy for older men with AV-LPC and less comorbidity are unclear. No randomized controlled trial has evaluated PC in men older than 80 years.

Studies have denoted the lack of consistency and robust results of the comparative output between curative-intended therapy, RP, and RT in older men with AV-LPC [19,20,21,22,23,24,25]. Some studies have reported no significant difference between the two modality approaches in terms of OS, biochemical recurrence, and cancer-specific survival [20,21]. However, another study found that RP caused more biochemical recurrence, which is a sign of increased risk of metastases and mortality [41]. Some studies have indicated that RP was related to a significant decrement in mortality rate compared with the RT [42,43], whereas another study demonstrated that massive-dose RT has distinguished biochemical outputs in both younger and older patients [44]. As a result, no definitive prime management exists for LPC, especially in older men with AV-LPC [42]. The inconsistent outcomes in comparing RP and RT in patients with PC might be caused by heterogeneity in patient risks, large age groups, and undefined multiple comorbidities; aforementioned risk classifications, age, and comorbidities are correlated with life expectancy [44]. In addition, no study has compared a specific RT modality, such as contemporary RT techniques of IMRT with higher irradiation dose and long-term (>18 months) ADT with RP in men with AV-LPC. Several meta-analyses and retrospective studies have reported that survival rates were poorer and mortality rates were higher among patients receiving RT than among those receiving RP [19,20,21,22,23,24,25,27]. For patients with AV-LPC, RP or RT with long-term ADT is the treatment of choice due to significant improvements in biochemical disease-free survival [45] and 10-year cancer-specific mortality [24]. Conventionally, RT has been deemed more suitable than RP for older men with PC. Our results, however, revealed that RP was better suited to IMRT + ADT in terms of OS, BF, LRR, and DM for older men with AV-LPC (Table 1, Table 2, Table 3 and Table 4 and Figure 1). Thus, our study addresses the issue of which curative-intent treatment is more efficacious for older men with AV-LPC.

Our study adopted the current risk categorization established by NCCN, which is typically distinct from the risk categorization by EAU [11]. For instance, the NCCN risk categorization merely applied grades 1–5 for PC in-lieu-of Gleason Scores, which originated from two sides; after that, appended jointly to provide the Gleason Score (within the range of 2–10). Thus, grade 4 was classified as the NCCN high-risk group and intermediate (4 + 3 = 7, EAU intermediate) risk group based on the EAU risk classification. Accordingly, patients categorized as the NCCN high-risk group PC were also the intermediate EUA categorization. However, such patients would be treated as covariates for the adjustments.

Our study has many strengths. First, there are limited data and a lack of consensus regarding the prime therapy for very old adults with AV-LPC. Our pioneering study on primary endpoints (all-cause mortality) and secondary endpoints (BF, LRR, and DM) allows physicians to compare oncological prognostication between RP and IMRT + ADT. Second, it is challenging to perform a trial including patients with HR-RP older than 80 years and to randomly choose RP or massive-dose IMRT plus long-term ADT. Our head-to-head PSM with a low selection bias and a long-term investigation of ≥5 years may address the vital issue in geriatric cancer medicine. Third, the covariates were also consistent between the RP and IMRT + ADT approaches, which alleviated the probability of bias and residual imbalance [46,47]. Most primary covariates were incorporated in the PSM analysis. The study findings will serve as a reference for guiding many parties, including physicians, patients, and the government, in selecting the most effectual and best treatment for older men with AV-LPC, associated with oncologic prognostication, quality of life, and surgical complications. These outputs of the study are crucial to geriatric and oncologic medicine as the number of these old patient groups with LPC is expected to increase in the future [9].

This study has several limitations. First, because we used data from the TCRD and NHIRD, we could not consider procedures that may have been carried out using out-of-pocket payments. However, few patients might have opted for it [48]. Second, because our cohort comprised Taiwanese people, our findings may have limited generalizability. Third, ICD-9 cm codes were set up to assess the presented diagnoses of whole comorbid circumstances, leaving room for error. However, the Taiwan Cancer Registry Administration irregularly conducts a review of diagrams and evaluations to confirm the accuracy of diagnosis and outlier charges and procedures; cases of malpractice or disparities are also severely penalized. Finally, information related to eating patterns or body mass index was not included in the study, which might also be risk factors for all-cause mortality [49]. The aforementioned constraints are implausible to predispose the inferences. Moreover, the present study is the first cohort study to include the whole population with the most up-to-date information and long-term investigation to evaluate the oncologic prognostication for older men with AV-LPC who have undergone RP versus massive-dose IMRT plus long-term ADT. Our data are expected to assist in stipulating health care policies and aiding physicians in choosing the optimal procedures for older men with AV-LPC.

## 5. Conclusions

This study determined the latest evidence on population-based large-scale data, specifically focused on very old patients with high-risk localized prostate cancer. In comparison with massive-dose IMRT plus long-term ADT, RP presented better long-term overall survival, as well as better all-cause mortality, biochemical recurrence, locoregional recurrence, and distant metastasis rates. Thus, RP should be preferred over IMRT + ADT in patients older than 80 years with AV-LPC. 

## Figures and Tables

**Table 1:** The AV-LPC statistics of all-cause death: Multivariate Cox regression model.

Covariates		Adjusted HR *	(95% CI)	*p* Value
Curative therapy	RP	ref		<0.0001
	IMRT + HT	2.00	(1.41–2.87)	
Dx year	2011–2012	ref		0.2369
	2013	0.75	(0.50–1.12)	
	2014	0.72	(0.45–1.20)	
	2015	0.98	(0.63–1.51)	
	2016	0.66	(0.41–1.09)	
CCI	0	ref		0.2483
	1	1.06	(0.54–1.21)	
	2+	1.32	(0.88–2.18)	
Congestive heart failure ailment		1.81	(0.67–3.01)	0.3510
Peripheral vascular related disorders		1.08	(0.233–1.44)	0.3901
Cerebrovascular problems		1.10	(0.75–1.57)	0.6651
Chronic pulmonary illness		1.24	(0.88–1.77)	0.2018
High blood sugar		1.10	(0.89–1.60)	0.3992
High blood pressure		1.01	(0.73–1.28)	0.8152
Wealth	<NTD 21,000	Ref		0.1209
	NTD 21,000–30,000	0.96	(0.50–1.19)	
	NTD 30,000–45,000	0.88	(0.67–1.30)	
	NTD 45,000+	0.76	(0.64–1.51)	
Hospital tier	Medical center	Ref		0.7856
	Others	1.03	(0.76–1.37)	
Hospital region	North	ref		0.4857
	Central	1.05	(0.68–2.11)	
	South	1.11	(0.78–1.61)	
	East	1.23	(0.50–1.34)	
AJCC T stage	T1	ref		0.4934
	T2a	1.05	(0.77–1.55)	
	T2b	1.13	(0.64–1.21)	
	T2c	1.27	(0.67–1.39)	
	T3a	2.01	(0.70–3.21)	
EAU risk classification	Localized—intermediate	ref		0.4622
	Localized—high	1.13	(0.76–1.54)	
	Locally advanced	1.66	(0.53–2.00)	

RP, radical prostatectomy; IMRT, intensity-modulated radiotherapy; HT, hormone therapy; HR, hazard ratio; CI, confidence interval; AJCC, American Joint Committee on Cancer; CCI, Charlson comorbidity index; T, tumor; NTD, New Taiwan Dollars. * Entire covariates referred in Table 1 were adjusted.

**Table 2:** The AV-LPC statistics of biochemical recurrence: Multivariate Cox regression model.

Covariates		Adjusted HR *	(95% CI)	*p* Value
Curative therapy	RP	ref		<0.0001
	IMRT + HT	1.77	(1.36–2.11)	
Dx year	2011–2012	ref		0.3367
	2013	0.97	(0.61–1.18)	
	2014	0.92	(0.61–1.21)	
	2015	0.90	(0.70–1.51)	
	2016	0.84	(0.80–1.66)	
CCI	0	Ref		0.5133
	1	1.09	(0.71–2.00)	
	2+	1.17	(0.68–2.10)	
Congestive heart failure ailment		1.04	(0.52–1.55)	0.7111
Peripheral vascular related disorders		1.03	(0.51–1.75)	0.7598
Cerebrovascular problems		1.02	(0.63–1.22)	0.3480
Chronic pulmonary illness		1.04	(0.60–1.32)	0.6791
High blood sugar		1.01	(0.50–1.21)	0.3110
High blood pressure		1.11	(0.49–1.20)	0.2608
Wealth	<NTD 21,000	ref		0.4510
	NTD 21,000–30,000	0.92	(0.75–1.18)	
	NTD 30,000–45,000	0.87	(0.61–1.22)	
	NTD 45,000+	0.73	(0.50–1.16)	
Hospital tier	Medical center	ref		0.2154
	Others	1.15	(0.91–1.33)	
Hospital region	North	ref		0.1321
	Central	1.00	(0.74–1.34)	
	South	1.03	(0.70–1.32)	
	East	1.17	(0.44–2.18)	
AJCC T stage	T1	ref		0.4317
	T2a	1.04	(0.81–1.50)	
	T2b	1.08	(0.71–1.55)	
	T2c	1.11	(0.54–1.87)	
	T3a	1.21	(0.52–1.98)	
EAU risk classification	Localized–intermediate	ref		<0.0001
	Localized–high	1.88	(1.45–2.41)	
	Locally advanced	2.03	(1.10–3.76)	

RP, radical prostatectomy; IMRT, intensity-modulated radiotherapy; HT, hormone therapy; HR, hazard ratio; CI, confidence interval; AJCC, American Joint Committee on Cancer; CCI, Charlson comorbidity index; T, tumor; NTD, New Taiwan Dollars. * adjusted for entire covariates referred to in Table 1.

**Table 3:** The AV-LPC statistics of locoregional recurrence: Multivariate Cox regression model.

Covariates		Adjusted HR *	(95% CI)	*p* Value
Curative therapy	RP	ref		<0.0001
	IMRT + HT	1.12	(1.04–1.33)	
Dx year	2011	ref		0.8034
	2012	0.96	(0.41–2.78)	
	2013	0.92	(0.49–2.80)	
	2014	0.97	(0.48–3.21)	
	2015	0.87	(0.46–3.71)	
CCI	0	ref		0.7607
	1	1.02	(0.43–2.41)	
	2+	1.10	(0.33–4.22)	
Congestive heart failure ailment		1.05	(0.47–1.24)	0.8033
Peripheral vascular related disorders		1.24	(0.44–3.63)	0.7963
Cerebrovascular problems		1.17	(0.60–3.06)	0.6972
Chronic pulmonary illness		1.28	(0.41–4.22)	0.6662
High blood sugar		1.11	(0.48–1.90)	0.3960
High blood pressure		1.30	(0.64–2.60)	0.4137
Wealth	<NTD 21,000	ref		0.8860
	NTD 21,000–30,000	1.14	(0.46–2.90)	
	NTD 30,000–45,000	1.09	(0.39–3.86)	
	NTD 45,000+	1.45	(0.40–4.88)	
Hospital tier	Medical center	ref		0.3308
	Others	1.09	(0.75–1.33)	
Hospital region	North	ref		0.4345
	Central	1.11	(0.59–2.97)	
	South	1.31	(0.96–4.01)	
	East	1.49	(0.71–5.01)	
AJCC T stage	T1	Ref		0.4252
	T2a	1.11	(0.61–2.20)	
	T2b	1.12	(0.57–2.04)	
	T2c	1.16	(0.71–2.70)	
	T3a	1.19	(0.88–5.01)	
EAU risk classification	Localized—intermediate	ref		<0.0001
	Localized—high	3.12	(1.22–4.82)	
	Locally advanced	3.18	(1.19–5.23)	

RP, radical prostatectomy; IMRT, intensity-modulated radiotherapy; HT, hormone therapy; HR, hazard ratio; CI, confidence interval; AJCC, American Joint Committee on Cancer; CCI, Charlson comorbidity index; T, tumor; NTD, New Taiwan Dollars. * adjusted for entire covariates referred in Table 1.

**Table 4:** The AV-LPC statistics on distant metastasis: Multivariate Cox regression model.

Covariates		Adjusted HR *	(95% CI)	*p* Value
Curative therapy	RP	ref		0.0311
	IMRT + HT	1.15	(1.06–1.71)	
Dx year	2011–2012	ref		0.8871
	2013	0.99	(0.50–1.61)	
	2014	0.92	(0.60–1.90)	
	2015	0.97	(0.62–2.06)	
	2016	0.93	(0.55–1.79)	
CCI	0	ref		0.4113
	1	1.15	(0.49–1.33)	
	2+	1.28	(0.71–2.52)	
Congestive heart failure ailment		1.12	(0.37–1.40)	0.2452
Peripheral vascular related disorders		1.09	(0.49–3.02)	0.6584
Cerebrovascular problems		0.56	(0.64–1.23)	0.1650
Chronic pulmonary illness		1.13	(0.65–1.65)	0.8109
High blood sugar		1.17	(0.51–1.63)	0.5064
High blood pressure		1.01	(0.61–1.41)	0.4878
Wealth	<NTD 21,000	ref		0.4862
	NTD 21,000–30,000	0.95	(0.66–1.80)	
	NTD 30,000–45,000	0.94	(0.72–2.31)	
	NTD 45,000+	0.86	(0.71–2.12)	
Hospital tier	Medical center	ref		0.4652
	Others	1.19	(0.53–1.44)	
Hospital region	North	ref		0.3094
	Central	1.03	(0.74–1.82)	
	South	1.05	(0.57–1.48)	
	East	1.16	(0.50–4.29)	
AJCC T stage	T1	Ref		0.2394
	T2a	1.19	(0.70–2.60)	
	T2a	1.23	(0.53–1.47)	
	T2c	1.51	(0.82–2.66)	
	T3a	1.75	(0.43–6.01)	
EAU risk classification	Localized–intermediate	ref		0.4708
	Localized–high	1.12	(0.70–2.82)	
	Locally advanced	1.29	(0.76–3.51)	

RP, radical prostatectomy; IMRT, intensity-modulated radiotherapy; HT, hormone therapy; HR, hazard ratio; CI, confidence interval; AJCC, American Joint Committee on Cancer; CCI, Charlson comorbidity index; T, tumor; NTD, New Taiwan Dollars. * adjusted for entire covariates referred in Table 1.

## Data Availability

The data sets supporting the study conclusions are included in this manuscript and its supplementary files.

## Supplementary Materials

The following supporting information can be downloaded at: https://www.mdpi.com/article/10.3390/jcm11123451/s1, Figure S1: Study flow-chart; Table S1: Propensity score–matched demographic and clinic characteristics of very old patients with NCCN high-risk prostate adenocarcinoma; Table S2: Multivariate Cox proportional hazards regression model analysis of cancer-specific mortaliyt of very old patients with high-risk prostate adenocarcinoma.

## Abbreviations

PC: prostate cancer; AV-LPC, high-risk localized prostate cancer; RP, radical prostatectomy; RT, radiotherapy; IMRT, intensity-modulated radiotherapy; aHR, adjusted hazard ratio; HR, hazard ratio; CI, confidence interval; DX, diagnosis; AUA, American Urological Association; EAU, European Association of Urology; NCCN, National Comprehensive Cancer Network; PSA, prostate-specific antigen; BF, biochemical failure; LRR, locoregional recurrence; DM, distant metastasis; PSM, propensity score matching; NHIRD, National Health Insurance Research Data; TCRD, Taiwan Cancer Registry Database; AJCC, American Joint Committee on Cancer; ADT, androgen deprivation therapy; ICD-9-CM, International Classification of Diseases, Ninth Revision, Clinical Modification; CCI, Charlson comorbidity index; T, tumor.
