# Supplementary material for: The Latest Data Specifically Focused on Long-Term Oncologic Prognostication for Very Old Adults with Acute Vulnerable Localized Prostate Cancer: A Nationwide Cohort Study
Source: J Clin Med. 2022 Jun 15;11(12):3451. doi: 10.3390/jcm11123451 (PMC9225393; doi:10.3390/jcm11123451)

Supplemental Table S1. Propensity score–matched demographic and clinic characteristics of very old patients with NCCN high-risk prostate adenocarcinoma.

		RP N = 277		IMRT+HT N = 382		
Covariates		n	(%)	n	(%)	p value
Age	Mean (SD)	84.6	(3.2)	85.1	(3.5)	0.1098
	Median (Q1–Q3)	84	(82–86)	85	(82–87)	
	80–89	256	(92.4)	340	(89.0)	0.9090
	90+	21	(7.6)	42	(11.0)	
Year of diagnosis	2011–2012	47	(17.0)	60	(15.7)	0.9837
	2013	49	(17.7)	72	(18.8)	
	2014	57	(20.6)	82	(21.5)	
	2015	62	(22.4)	83	(21.7)	
	2016	62	(22.4)	85	(22.3)	
CCI scores	0	102	(36.8)	145	(38.0)	0.7626
	1	77	(27.8)	104	(27.2)	
	2+	98	(35.4)	133	(34.8)	
Myocardial infarction		4	(1.4)	7	(1.8)	0.8348
Congestive heart failure		10	(3.6)	13	(3.4)	0.7740
Peripheral vascular disease		10	(3.6)	10	(2.6)	0.4913
Cerebrovascular disease		31	(11.2)	48	(12.6)	0.8997
Chronic pulmonary disease		41	(14.8)	57	(14.9)	0.7090

		RP N = 277		IMRT+HT N = 382		
Covariates		n	(%)	n	(%)	p value
Diabetes		73	(26.4)	94	(24.6)	0.6732
Hypertension		166	(59.9)	233	(61.0)	0.9782
Income	<NTD 21,000	92	(33.2)	119	(31.2)	0.8053
	NTD 21,000–30,000	124	(44.8)	170	(44.5)	
	NTD 30,000–45,000	29	(10.5)	41	(10.7)	
	NTD 45,000+	32	(11.6)	52	(13.6)	
Hospital area	North	128	(46.2)	169	(44.2)	0.9637
	Central	82	(29.6)	115	(30.1)	
	South	61	(22.0)	89	(23.3)	
	East	6	(2.2)	9	(2.4)	
Hospital level	Medical center	178	(64.3)	244	(63.9)	0.5094
	Others	99	(35.7)	138	(36.1)	
AJCC T stage	T1	96	(34.7)	128	(33.5)	0.9366
	T2a	57	(20.6)	77	(20.2)	
	T2b	26	(9.4)	44	(11.5)	
	T2c	89	(32.1)	120	(31.4)	
	T3a	9	(3.2)	13	(3.4)	
Gleason score	≤5	0		0		0.9570
	6	19	(6.9)	33	(8.6)	

Covariates		RP N = 277 n	(%)	IMRT+HT N = 382 n	(%)	p value
	7	187	(67.5)	253	(66.2)	
	8	44	(15.9)	64	(16.8)	
	9+	22	(7.9)	23	(6.0)	
	Missing	5	(1.8)	9	(2.4)	
Grade group (max of Gleason grade)	1–2	5	(1.8)	9	(2.4)	0.9624
	3	19	(6.9)	32	(8.4)	
	4	223	(80.5)	304	(79.6)	
	5	30	(10.8)	37	(9.7)	
PSA, ng/mL	Mean (SD)	11.7	(7.6)	12.7	(11.6)	0.1942
	Median (IQR, Q1–Q3)	10.3	(6.8–15.1)	10.5	(7.2–14.9)	
	0–5	22	(7.9)	34	(8.9)	0.9770
	5–10	95	(34.3)	116	(30.4)	
	10–20	109	(39.4)	158	(41.4)	
	20+	15	(5.4)	28	(7.3)	
	Missing	36	(13.0)	46	(12.0)	
EAU risk classification	Localized—intermediate	125	(45.1)	171	(44.8)	0.8521
	Localized—high	143	(51.6)	198	(51.8)	
	Locally advanced	9	(3.2)	13	(3.4)	
Follow-up time, months	Mean (SD)	61.7	(18.4)	58.4	(18.9)	0.8742

Covariates	RP N = 277 n	(%)	IMRT+HT N = 382 n	(%)	p value
All-cause death	26	(9.4)	76	(19.9)	0.0003
Cancer-specific mortality	20	(7.2)	41	(10.7)	0.0356
Biochemical recurrence	52	(18.8)	130	(34.0)	<0.0001
Locoregional recurrence	12	(4.3)	20	(5.2)	0.0468
Distant metastasis	25	(9.0)	43	(11.3)	0.3639

RP, radical prostatectomy; RT, radiotherapy; IMRT, intensity-modulated radiotherapy; HT, hormone therapy; NCCN, National Comprehensive Cancer Network; PSA, prostate-specific antigen; AJCC, American Joint Committee on Cancer; CCI, Charlson comorbidity index; T, tumor; NTD, New Taiwan Dollars; SD, standard deviation; IQR, interquartile range

Supplemental Table S2. Multivariate Cox proportional hazards regression model analysis of cancer-specific mortaliyt of very old patients with high-risk prostate adenocarcinoma.

**Covariates**		**Adjusted HR ***	**(95% CI)**	**p value**
Curative treatment	Radical prostatectomy	ref		0.0411
	IMRT + HT	1.04	(1.01–1.21)	
Year of diagnosis	2011-2012	ref		0.7809
	2013	0.91	(0.51–1.52)	
	2014	1.11	(0.61–1.94)	
	2015	1.13	(0.66–2.00)	
	2016	0.91	(0.50–1.72)	
CCI scores	0	ref		0.2415
	1	0.88	(0.47–1.27)	

Covariates		Adjusted HR *	(95% CI)	p value
	2+	1.11	(0.70–2.20)	
Congestive heart failure		0.77	(0.43–1.40)	0.2356
Peripheral vascular disease		0.70	(0.36–2.97)	0.5251
Cerebrovascular disease		0.74	(0.38–1.21)	0.2391
Chronic pulmonary disease		1.05	(0.64–1.76)	0.8170
Diabetes		0.96	(0.65–1.74)	0.7802
Hypertension		0.90	(0.77–1.60)	0.5931
Income	<NTD 21,000	ref		0.6040
	NTD 21,000–30,000	0.96	(0.73–1.77)	
	NTD 30,000–45,000	0.90	(0.77–2.57)	
	NTD 45,000+	0.86	(0.70–2.27)	
Hospital level	Medical center	ref		0.6082
	Others	1.01	(0.66–1.41)	
Hospital area	North	ref		0.5140
	Central	1.04	(0.68–1.31)	
	South	1.06	(0.58–1.33)	
	East	1.22	(0.69–4.01)	
AJCC T stage	T1	ref		0.2741
	T2a	1.09	(0.85–1.66)	
	T2b	1.15	(0.69–1.47)	
	T2c	1.22	(0.88–2.76)	
	T3a	1.33	(0.42–5.90)	
EAU risk classification	Localized–intermediate	ref		0.5817
	Localized–high	1.29	(0.70–2.07)	
	Locally advanced	1.87	(0.59–3.57)	

RP, radical prostatectomy; RT, radiotherapy; IMRT, intensity-modulated radiotherapy; HT, hormone therapy; aHR, adjusted hazard ratio; HR, hazard ratio; CI, confidence interval; AJCC, American Joint Committee on Cancer; CCI, Charlson comorbidity index; T, tumor; NTD, New Taiwan Dollars.

*adjusted for all covariates mentioned in Table 1.

Supplemental Figure S1. Study flow-chart.

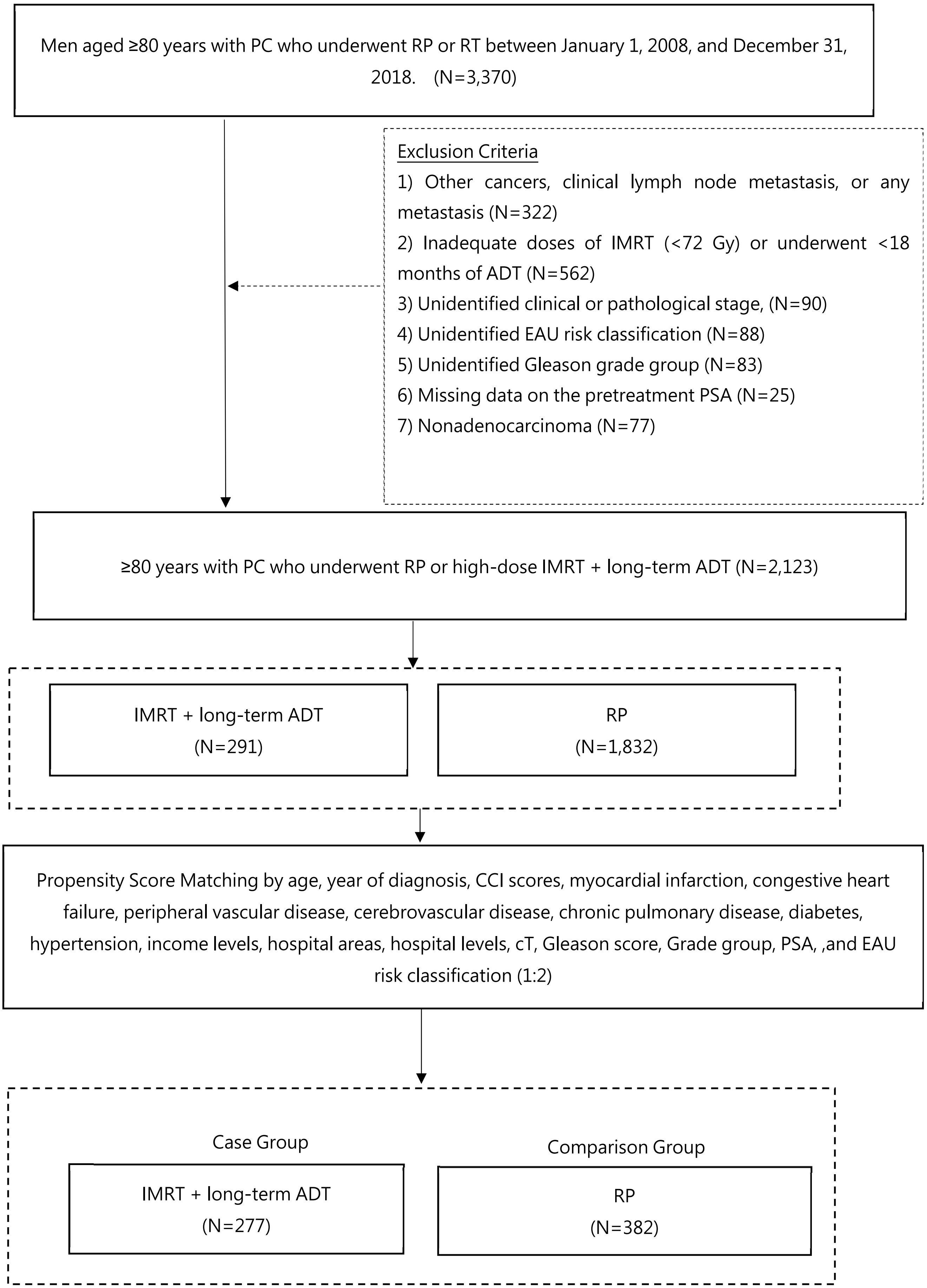

Supplement: Supplementary file 1 [file jcm-11-03451-s001.zip › jcm-1740415-supplementary.pdf]
